# Sense of Coherence and Personality Traits Related to Depressive State

**DOI:** 10.1155/2014/738923

**Published:** 2014-10-09

**Authors:** Yoko Kikuchi, Makoto Nakaya, Miki Ikeda, Shoko Okuzumi, Mihoko Takeda, Miyoko Nishi

**Affiliations:** ^1^Department of Psychiatry, Musashino Red Cross Hospital, Kyounancho 1-26-1, Musashino, Tokyo 180-8610, Japan; ^2^Faculty of Medicine, Tokyo Medical and Dental University, Yushima 1-5-45, Bunkyouku, Tokyo 113-8570, Japan; ^3^Department of Nurse, Musashino Red Cross Hospital, Kyounancho 1-26-1, Musashino, Tokyo 180-8610, Japan

## Abstract

*Aims*. The current study aims to examine the influence of job stress, SOC, and personality traits on depressive state. *Methods*. A self-reported survey was conducted among 347 female nurses in a general hospital. Job stress was measured using the Japanese version of the Brief-Job Stress Questionnaire scale. Depressive state was assessed by the K6 scale. We used 13-item SOC scale. Personality traits were assessed by the Japanese version of Ten-Item Personality Inventory. Multiple liner regression analyses were conducted to examine predictors that significantly affect depressive state. *Results*. Job and life satisfaction and SOC negatively related to the depressive state (*β* = −0.76, *P* < 0.01; *β* = −0.18, *P* < 0.001, resp.) while neuroticism was positively correlated (*β* = 0.49, *P* < 0.001). Also, intrinsic rewards tended to negatively relate (*β* = −0.80, *P* < 0.1). *Conclusions*. From a practical perspective, the possible influence of SOC and neurotic personality on depressive state should be considered for health care professionals.

## 1. Introduction

Job stress and personality traits are known to be significant determinants of depressive state. Previous studies found that, in hospital staff members, job stress may adversely affect mental health and lead to depressive symptoms [1–8]. In female nurses at a general hospital, our group found a significant relationship between depressive state and job stress measured by the effort-reward imbalance model [6]. A meta-analytic review of Stansfeld [9] suggested that job stresses were prospective risk factors for depressive and anxiety disorder. Personality aspects were suggested to play an important role for medical symptoms, work satisfaction, and work stress. Bienvenu et al. [10] investigated the relationship between personality aspects and depressive state in subjects examined by psychiatrists with the Schedules for Clinical Assessment and the Revised NEO Personality Inventory; they showed that depressive and anxiety disorders were associated with high neuroticism. We also previously investigated the relationship between job stress and temperaments in female nurses and suggested that nurses with depressive or anxious temperaments should be identified and monitored for signs of job stress [11].

One measurement that is inversely correlated with depressive state is the sense of coherence (SOC), proposed by Antonovsky [12]. SOC is based on the salutogenic model of health [13, 14] and is composed of three factors: comprehensibility, manageability, and meaningfulness. The comprehensibility factor suggests that stimuli deriving from internal and external environments are structured, predictable, and explicable, while manageability assesses whether resources are available to meet the demands posed by these stimuli. The third factor, meaningfulness, indicates if such demands are challenges, worthy of investment and engagement. The SOC is a personality dimension hypothesized to influence stress recognition style, to facilitate stress management, and to contribute to overall well-being. Previous studies suggested that the SOC modified the effect of job stress on mental health [15–20] and that a weak SOC was a strong predictor of mental distress including depressive state [16, 17, 19, 20]. Both personality traits and SOC are thought to influence the effects of job stress on depressive state, but the nature and relative strength of these relationships have not been established. The aim of the current study is to examine the influences of job stress, SOC, and personality traits on depressive state.

## 2. Materials and Methods 

Study subjects consisted of nurses (*n* = 740) at a general hospital with 611 beds in an urban area of Japan. Specialties of the nurses included intensive care, pediatrics, surgery, oncology, and emergency medicine. The questionnaire was distributed to subjects by their supervisors in December 2013. Management allowed the nurses to complete the questionnaires during their shifts. An explanation of the nature of the survey accompanied the questionnaire, which was anonymous and voluntary. Consent was assumed if participants answered the questionnaire. The study was approved by both the general hospital's board of directors and its Committee for the Prevention of Physical Disease and Mental Illness among Health Care Workers.

The questionnaire collected data on age, job rank (manager, middle manager, or staff nurse), hours of work (full time or part time), shift work, and overtime. Overtime was reported in hours per week. Overtime was voluntary but limited to 45 hours per month. Shift work categories included “no shift work,” “shift work with night shift rotations,” or “shift work without night shift rotations.” Only managers have a choice in shift assignment.

We measured job stress using the Japanese version of the Brief-Job Stress Questionnaire (BJSQ), with three subscales: job stressors (17 items), stress responses (29 items), and social supports as buffering factors (9 items). The BJSQ also measured job satisfaction and life satisfaction. The questionnaire contains 57 items with four-point Likert-type responses (from “agree” = 4 to “disagree” = 1). It has been used widely established as a method for assessing job stress in Japan and validated and tested for reliability [21].

The SOC scale consisted of 13 items assessing comprehensibility, manageability, and meaningfulness. Each item was scored with Likert-type responses from 1 to 7 points; the total of item scores was calculated as the SOC score. Higher scores indicate stronger SOC.The scale has been validated and tested for reliability [22].

We assessed depressive state using the K6 short screening questionnaires developed in accordance with the World Health Organization (WHO) translation guidelines [23]. The K6 consists of six items on depression and anxiety, measured on a 5-point scale (0–4). Higher scores indicate more severe depressive state. The K6 was translated into Japanese and showed good validity to DSM-IV mood and anxiety disorders in a community sample.

We measured personality traits using the Japanese version of the Ten-Item Personality Inventory (TIPI-J). TIPI-J is a measure of the Big-Five personality dimensions: extraversion, agreeableness, conscientiousness, emotional stability, and openness to experience [24]. Each item was scored at 1 to 7 points and the scores were totaled. It has been validated and tested for reliability in Japan by Oshio et al. [25].

We checked the distributions of each variable and some of them had nonnormal distributions. Then we used Spearman's correlation analyses to investigate potential relationships among work environment, age, job stress, SOC, personality traits, and depressive state. We examined the relationships between depressive state and predictors using multiple linear regression analysis. We added variables in 3 different steps to see the influence of additional variables. We considered job stress, SOC, and personality traits as independent variables and depressive state as an explanatory variable. In Step 1, age, job stress, and work-related variables that showed a significant association with depressive state were added to the equations of depressive state. In Step 2, we added SOC scores; in Step 3, we added personality variables.

We used SPSS11 (SPSS Inc., Chicago, IL, USA) for all analyses.

## 3. Results

Characteristics of the study respondents are shown in Table 1. Completed questionnaires were returned by 386 out of 740 nurses (response rate, 52.2%). Male nurses were excluded from the analysis due to a low sample size; only 20 of the 42 (47.6%) male nurses responded. Subjects with missing values for job stress, personality inventory, and SOC were also excluded (*n* = 19). The final sample for analysis consisted of 347 female nurses (47.0%), including managers and middle managers. Mean scores on each subscale of the questionnaire are shown in Table 1.

Age was negatively correlated with depressive state but positively related to SOC. SOC showed a relationship with shift work, job rank, and overtime hours. For almost all job stressors and personality traits, SOC and depressive state showed inverse correlation patterns. There were significant relationships between SOC and all measured personality traits; above all, neuroticism had the strongest correlation (Table 2).

Results of the multiple linear regression analyses for depressive state are shown in Table 3. Age, overtime hours per week, intrinsic rewards, job satisfaction, and life satisfaction explained 39% of the variance in depressive state. In Step 2, SOC accounted for an additional 11% (*F* = 19.63, *P* < 0.001) of the variance. In Step 3, personality variables accounted for an additional 3% (*F* = 16.56, *P* < 0.001) of the variance. Job and life satisfaction and SOC negatively related to the depressive state (*β* = −0.76, *P* < 0.01; *β* = −0.18, *P* < 0.001, resp.,) while neuroticism was positively correlated (*β* = 0.49, *P* < 0.001). Also, intrinsic rewards tended to negatively relate (*β* = −0.80, *P* < 0.1). Those beta-value means that, for example, a change of = −0.76 units on the job and life satisfaction increase depressive state by one unit, SOC had the strong correlation with depressive state (*r* = −0.67), but the expected collinearity did not occur in the regression model.

## 4. Discussion

We found that SOC, neuroticism, and job satisfaction were predictors of depressive state. Specifically, SOC was mainly associated with depressive state among female nurses.

The strength of our study is the investigation of the association among personality traits, SOC, and depressive state. To our knowledge, there was no previous study that investigated the nature and the relative strength of these relationships.

The association between SOC and depressive state was in accordance with previous studies. Malinauskiene et al. [17] indicated that weak SOC was a strong predictor of the GHQ totals among nurses in Lithuania (adjusted  OR = 4.11; 95% CI 2.24–7.56). Urakawa et al. [20] found that SOC was inversely associated with depression in Japanese factory workers (*β* = −0.41*P* < 0.001; *R* ^2^ = 0.34). Among Japanese resident doctors, poorer mental health status was associated with weaker SOC scores [16]. Hospital staff members with stronger SOC may cope better with their job stress and thereby prevent the depressive state.

In our study, SOC was correlated with older age (Table 2), consistent with previous studies [12, 17, 20]. For example, Urakawa et al. [20] found that the high SOC score group were older compared with the low SOC score group. These findings support the view that SOC is not inborn or innate but is a learned capacity. Nurses with higher SOC scores are also more satisfied with their job and life (Table 2). Antonovsky [12] posited that a person with a higher SOC score would be less likely to perceive many stressful situations as threatening and anxiety provoking, whereas a person with a lower SOC score will be more vulnerable to various stressors in his or her life. Intervention support enhancing the SOC could be effective to prevent nurses from entering a depressive state.

The present study has some drawbacks. First, the sample size was a relatively small number of participants with a low response rate in only one general hospital. A longitudinal study of a larger sample is necessary. Second, we only examined work-related factors. Malinauskiene et al. [26] indicated that life-threatening events, such as divorce, family financial crisis, or death of a first-degree relative or close friend, were associated with negative subjective health among hospital nurses. Other studies found associations between life-threatening events and poor mental health and between marriage and SOC [20]. Social support from family may be important for developing SOC. In addition, characteristics of private life should be considered in evaluating general health perspectives of female employees; these nonoccupational factors should be included in the questionnaires for futures studies. Third, according to Antonovsky [12], a person with a very high score of SOC was hypothesized to present inflexibility, called “rigid SOC.” However, there has been no evidence of a cutoff point indicating “rigid SOC.” Potential negative effects of higher SOC score on mental health should be investigated in the future. Antonovsky [12] found that SOC was not influenced by temperament or personality traits. These contradictions, along with the current findings on the neurotic personality trait, indicate a need for future investigation of the relationships between personality traits and SOC.

## 5. Conclusions

In conclusion, SOC, neurotic personality, and job satisfaction ratings were related to the depressive state among female nurses in the Japanese general hospital. Our findings provide insight into factors associated with depressive state among nurses. From a practical perspective, the influence of SOC and neurotic personality on depressive state should be considered for health care professionals. Intervention support such as group cognitive psychotherapy to strengthen comprehensibility, manageability, and meaningfulness may help people cope better with job stress and may reduce the risk of depressive state in nurses.

## Figures and Tables

**Table 1:** Characteristics of the subjects (total *N* = 347).

	*n* (%)			
*Work-related variables *				
Hours of work				
Full time	327 (94)			
Part time	17 (5)			
Shift work				
Rotation to nights	266 (77)			
No rotation to nights	12 (4)			
No shift work	68 (20)			
Job rank				
Manager	18 (5)			
Middle manager	25 (7)			
Staff nurse	301 (87)			

	Mean	SD	Max.	Min.

Age	33.7	9.2	21	62
Overtime per week	7.0	5.4	0	40
Job stressors				
Quantitative job overload	9.9	1.7	5	12
Qualitative job overload	10.3	1.5	6	12
Physical demands	3.3	0.8	11	40
Job control	6.8	1.7	3	10
Skill utilization	3.2	0.6	1	4
Interpersonal conflict	6.3	1.8	3	12
Poor physical environment	2.4	0.8	1	4
Suitable jobs	2.7	0.7	1	4
Intrinsic rewards	3.0	0.7	1	4
Psychological distress or mood				
Vigor	5.9	2.2	3	12
Anger, irritability	7.2	2.4	3	12
Fatigue	8.4	2.7	3	12
Anxiety	7.0	2.6	3	12
Depression	12.1	4.8	6	24
Buffering factors				
Supervisor support	7.4	2.1	3	12
Coworker support	8.6	1.9	5	12
Family and friends support	10.0	1.9	3	12
Job satisfaction and life satisfaction	5.3	1.3	2	8
Sense of coherence	54.2	11.9	16	87
Personality				
Extraversion	8.5	2.8	2	14
Agreeableness	9.7	1.9	4	14
Conscientiousness	7.4	2.4	2	14
Neuroticism	8.4	2.5	2	14
Openness	7.3	2.2	2	14

**Table 2:** Spearman's correlation between depression, personality, sense of coherence, and job stress.

	Depression	*P* value		SOC	*P* value	
Age	−0.18	0.00		0.27	0.00	
Hours of work	−0.03	0.55	n.s	0.10	0.08	n.s
Shift work	0.09	0.08	n.s	−0.16	0.00	
Job rank	0.05	0.36	n.s	−0.14	0.01	
Overtime per week	0.34	0.00		−0.33	0.00	
Job stressors						
Quantitative job overload	0.34	0.00		−0.34	0.00	
Qualitative job overload	0.26	0.00		−0.28	0.00	
Physical demands	0.23	0.00		−0.25	0.00	
Job control	−0.29	0.00		0.36	0.00	
Skill utilization	−0.07	0.19	n.s	0.11	0.04	
Interpersonal conflict	0.22	0.00		−0.28	0.00	
Poor physical environment	0.12	0.03		−0.12	0.03	
Suitable jobs	−0.33	0.00		0.40	0.00	
Intrinsic rewards	−0.33	0.00		0.33	0.00	
Buffering factors						
Supervisor support	−0.27	0.00		0.35	0.00	
Coworker support	−0.23	0.00		0.27	0.00	
Family and friends support	−0.13	0.01		0.16	0.00	
Job satisfaction and life satisfaction	−0.44	0.00		0.47	0.00	
Sense of coherence	−0.67	0.00				
Personality						
Extraversion	−0.19	0.00		0.17	0.00	
Agreeableness	−0.19	0.00		0.30	0.00	
Conscientiousness	−0.17	0.00		0.27	0.00	
Neuroticism	0.45	0.00		−0.49	0.00	
Openness	−0.16	0.00		0.20	0.00	

**Table 3:** Multiple linear regression for depressive state.

Independent variables	Step 1			Step 2			Step 3		
	Unstandardized *β*	95% CI	*P* value	Unstandardized *β*	95% CI	*P* value	Unstandardized *β*	95% CI	*P* value
Age	−0.10	−0.17–−0.03	0.004	−0.04	−0.04–0.03	0.247	−0.02	−0.08–0.04	0.486
Overtime per week	0.15	0.04–0.26	0.008	0.08	0.08–0.05	0.121	0.07	−0.03–0.17	0.156
Job stressors									
Quantitative job overload	0.27	−0.19–0.73	0.249	0.16	0.16–0.21	0.453	0.12	−0.30–0.53	0.583
Qualitative job overload	0.29	−0.18–0.77	0.224	0.09	0.09–0.22	0.699	0.06	−0.36–0.48	0.784
Physical demands	0.10	−0.71–0.91	0.807	0.15	0.15–0.37	0.693	0.18	−0.54–0.89	0.624
Job control	−0.17	−0.57–0.22	0.393	−0.11	−0.11–0.18	0.563	−0.07	−0.42–0.28	0.699
Interpersonal conflict	0.09	−0.27–0.45	0.632	−0.03	−0.03–0.17	0.854	0.03	−0.29–0.35	0.856
Poor physical environment	0.43	−0.26–1.13	0.218	0.39	0.39–0.32	0.223	0.47	−0.14–1.09	0.132
Suitable jobs	−0.75	−1.79–0.29	0.157	−0.25	−0.25–0.48	0.603	−0.09	−1.03–0.85	0.854
Intrinsic rewards	−0.94	−1.96–0.07	0.069	−0.63	−0.63–0.47	0.179	−0.80	−1.72–0.12	0.088
Buffering factors									
Supervisor support	−0.13	−0.36–0.33	0.939	0.09	0.09–0.16	0.570	0.11	−0.20–0.42	0.492
Coworker support	−0.12	−0.48–0.23	0.490	−0.08	−0.08–0.16	0.624	−0.10	−0.41–0.22	0.559
Family and friends support	−0.11	−0.42–0.20	0.497	0.04	0.04–0.14	0.786	0.05	−0.23–0.33	0.727
Job satisfaction and life satisfaction	−1.34	−1.86–−0.81	<0.001	−0.77	−0.77–0.25	0.003	−0.76	−1.25–−0.28	0.002
Sense of coherence				−0.22	−0.22–0.03	<0.001	−0.18	−0.24–−0.12	<0.001
Personality									
Extraversion							−0.04	−0.23–0.15	0.667
Agreeableness							0.09	−0.21–0.39	0.553
Conscientiousness							−0.02	−0.23–0.19	0.850
Neuroticism							0.49	0.26–0.72	<0.001
Openness							−0.03	−0.31–0.15	0.492

*R*	0.62			0.71			0.73		
*R* ^ 2^	0.39			0.50			0.53		
*F*	13.45		<0.001	19.63		<0.001	16.56		<0.001
